# Prediction of Residual Life of In-Service P91 Steel Joints Based on Fracture Behavior

**DOI:** 10.3390/ma17122789

**Published:** 2024-06-07

**Authors:** Kai Yan, Yongjiang Cai, Denghui Wang, Shichao Zhang, Shuang Yi, Fulai Yang, Zheng Zhang

**Affiliations:** 1 China Special Equipment Inspection & Research Institute, Beijing 100029, China; yankai@csei.org.cn; 2 Guangdong Honghaiwan Power Generation Co., Ltd., Shanwei 516623, China; ydsw_cyj@163.com; 3 School of Materials Science and Engineering, Beihang University, Beijing 100191, China; zhangshichaomail@163.com (S.Z.); yishuang@buaa.edu.cn (S.Y.); buaayangfulai@163.com (F.Y.); zhangzh@buaa.edu.cn (Z.Z.)

**Keywords:** in-service P91 steel joints, remaining life prediction, Larson–Miller method, type IV cracking

## Abstract

P91 steel and P91 steel joints experience performance degradation after serving for 30,000 h in working conditions. To clarify the damage and failure behavior and remaining life of the joints during subsequent service, further creep testing was conducted on the welded joints of P91 steel that had been in service for 30,000 h at three temperatures: 550 °C, 575 °C, and 600 °C. The fracture surface and the cross-section damage behavior were characterized by SEM and EBSD methods. The results show that there are two types of fracture modes in the joints at different temperatures: ductile cracking occurring at the BM, and type IV cracking occurring in the FGHAZ. The threshold stress for fracture mode transition decreases with an increase in working temperature. Type IV cracking near the HAZ is the main reason for the premature failure of joints during service. And based on the fracture mode, the dual-constant L-M method was proposed to predict the strength of in-service joint materials. The testing data are discussed and classified based on the fracture mode in this method, which has high accuracy and can prevent the premature failure of joints.

## 1. Introduction

SA335/SA335M P91 steel is an improved 9Cr-1Mo high-strength martensitic heat-resistant steel, which was developed by Oak Ridge National Laboratory and Combustion Engineering in the late 1970s [1]. P91 steel is widely used to manufacture critical components in ultra-supercritical (USC) power plants due to their high thermal conductivity, low thermal expansion coefficient, high persistent strength, and stable high-temperature creep resistance [2,3,4]. As a part of the P91 steel pipeline, welded joints have a complex microstructure and mechanical properties, and are the weakest area in the main body of the pipeline. Since the first batch of P91 heat-resistant steel pipes was put into use, there have been several accidents caused by the premature failure of joints [5,6,7].

The welding process of large steam pipelines and other components in boiler units adopts the fusion welding method. During the welding process, multiple heat inputs have an impact on the microstructure of the welded metal and the base metal on both sides, changing its structural characteristics. Even if corresponding normalization and tempering treatments are carried out in the later stage to improve the toughness of the joint, the degradation of microstructures and mechanical properties under long-term high temperature and high pressure cannot be avoided. The degradation of joints often leads to different failure behaviors.

**Figure 1:**
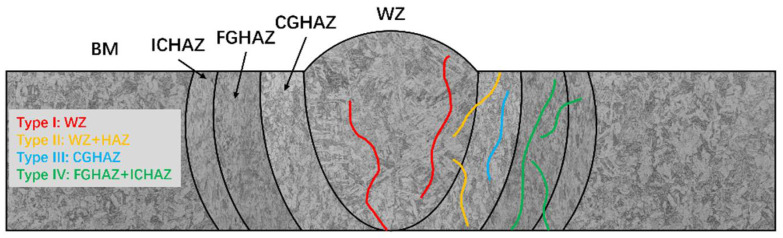
Schematic diagram of four cracking behaviors of joints.

Based on the location of cracks, the cracking behavior of joints can be divided into four types [8], as shown in Figure 1, where type I cracking is limited to the weld metal, i.e., within the weld nugget zone (WZ); type II cracking occurs in the WZ and extends laterally towards the base metal (BM); type III cracking occurs within the coarse-grained heat-affected zone (CGHAZ); type IV cracking occurs in the fine-grained heat-affected zone (FGHAZ) and critical heat-affected zone (ICHAZ) far from the WZ [9,10]. Some results show that type IV cracking is a prominent problem in the long-term service of martensitic heat-resistant steel, which is a brittle fracture behavior. The type IV cracking of the joint will significantly reduce the service life of the components, and there is no obvious deformation before the fracture occurs, seriously threatening the operational safety of the unit [5,9].

Due to the different grain structures, precipitated phase distributions, and mechanical properties of each zone of welded joints, the creep deformation behavior of joints is complex under the action of high temperature and stress. T. Sakthivel [11] found that during the creep process of P92 steel welded joints at a temperature of 923 K, the fracture position gradually changed from ductile fractures at the base metal to brittle fractures in the FGHAZ of the joint, that is, type IV cracking, and believed that the cause of type IV cracking was the formation of the Laves phase in the FGHAZ and a reduction in material strength caused by the rapid roughening speed, which led to the premature failure of the joints. Zhang [12] et al. found the same behavior in a study of G115 welded joints and believed that the reason for the change in the fracture position was that with a decrease in stress, the damage mechanism changed from martensitic lath cracking to microstructure degradation. Wang [13] et al. found that the fracture mode shifted from the WZ to FGHAZ with a decrease in stress and did not fail from the P92 BM, and the failure behavior of the joint was related to the matching principle of the weldment and the welding consumables under high stress.

At present, there is no unified conclusion on failure modes in research, and there are few reports on the prediction of this behavior. Traditional creep life prediction methods cannot accurately predict the creep life of in-service heat-resistant steel joints. As is well known, there is a difference between research on the microstructure and performance evolution of heat-resistant steel welded joints under laboratory conditions and engineering practice, which is the main reason for the difficulty in reproducing the early failure behavior (type IV cracking) of joints in the laboratory. Based on the study of the creep failure behavior of P91 steel welded joints after 33,000 h of service, this paper verifies the failure behavior that may occur in the long-term service of welded joints. This study solves the problem of difficulty in generating actual damage and failure under laboratory conditions, providing a reference for studying the real damage and failure behavior of heat-resistant steel joints. The traditional Larson–Miller method has been modified to provide a method for predicting the service of joints under working conditions, which can provide guidance for improving the efficiency of in-service components and ensuring their safety.

## 2. Experiment

### 2.1. Materials

P91 heat-resistant steel and its welded joints used in this study come from the reheat steam hot-end pipeline of a power plant that has been in service for 33,000 h, with a diameter of Φ559 mm, a wall thickness of 30 mm, and a joint made with Tungsten Inert Gas Welding (TIG) backing and filling with Shielded Metal Arc Welding (SMAW). The filling material models are ER90S-B9 and E9015-B9 (Materials provided by the Guangdong Honghaiwan Power Generation Co., Ltd., Shanwei, China), respectively, and the shielding gas is argon. After welding, it is tempered at 760 °C for 3 h. Its early working condition service temperature is 571 °C, and the working stress is 25.4 MPa.

### 2.2. Creep Tests

The creep test of welded joints adopts the RDJ-30 mechanical high-temperature creep testing machine provided by China Special Equipment Inspection & Research Institute, Beijing, China. The creep specimen is in the shape of a rod, with a gauge section diameter of 16 mm and a length of 80 mm. The joint is in the middle of the gauge section, and the heat-affected zone and base metal are symmetrical about the center of the weld zone. Creep tests were conducted at three temperatures, 550 °C, 575 °C, and 600 °C, with stress ranges ranging from 155 to 230 MPa, 120 to 190 MPa, and 85 to 160 MPa, respectively. The test atmosphere was air. The creep tests at three different temperatures were all stopped after rupture, and the test duration was between 100 and 15,000 h.

### 2.3. Microstructure Characterization

The hardness of the joint before and after the creep test was determined using an Enoli FALCON511 microhardness tester provided by Beihang university, Beijing, China, the test force was 500 gf, the holding time was 15 s, and the distance between adjacent indentations was 0.15 mm. To confirm the distribution of joint hardness, the hardness cloud map was measured using a matrix of 120 × 400 for a total of 48,000 points. The hardness test of the fracture section after creep tests was performed at the medium-thickness position. The creep fracture surfaces were observed by a stereo microscope, and the microstructure of each area of the fracture was cleaned by boiling in a NaOH + KMnO_4_ + H_2_O water bath for 120 min, ultrasonic cleaning with saturated oxalic acid solution for 8 min, ultrasonic cleaning with acetone solution for 8 min, and repeated pasting and brushing with tape and a brush, and the microstructure of the fracture was characterized by a JSM6010 scanning electron microscope (SEM). The cross-section of the fracture was polished with sandpaper, a ferric chloride solution was used for etching (ferric chloride 10 g + hydrochloric acid 40 mL + water 40 mL), and the microstructure was characterized by the JSM7500 SEM provided by Beihang university, Beijing, China. The texture and misorientation angle distribution of the samples at different positions were investigated by electron back-scattered diffraction (EBSD, JSM-F100, Orion) for characterization at a step size of 0.4 μm and a voltage of ~20 kV.

## 3. Results and Discussions

### 3.1. Materials before Testing

**Figure 2:**
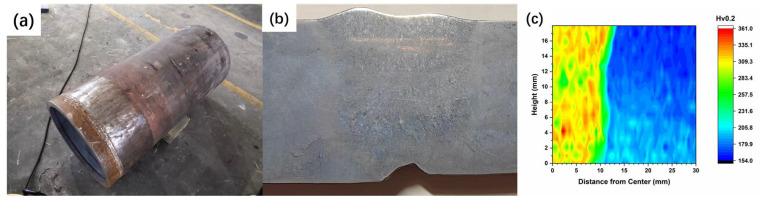
Experimental materials and hardness distribution: (**a**) reheat steam hot section pipeline; (**b**) cross-section of a welded joint; (**c**) hardness cloud map.

The composition and macroscopic morphology of P91 steel welded joints after 33,000 h of service are shown in Table 1 and Figure 2. In Table 1, BM represents the base metal of P91 steel welded joints, and WZ represents the weld zone.

There is not much difference between the chemical composition of WZ and BM. The results indicate that the main components of the WZ and BM meet the standard requirements during the service, and the proportion of different elements does not change significantly. The hardness distribution cloud map in Figure 2c shows that the welded joints after long-term service still have high-strength matching characteristics. The hardness of WZ is much higher than BM, and the hardness of the HAZ is located between the WZ and BM, showing a gradient decrease from WZ to BM. The hardness of the outer wall part of the BM has decreased the most and no longer meets the minimum requirements for standard hardness.

### 3.2. Fracture Behavior of Creep Specimens

**Figure 3:**
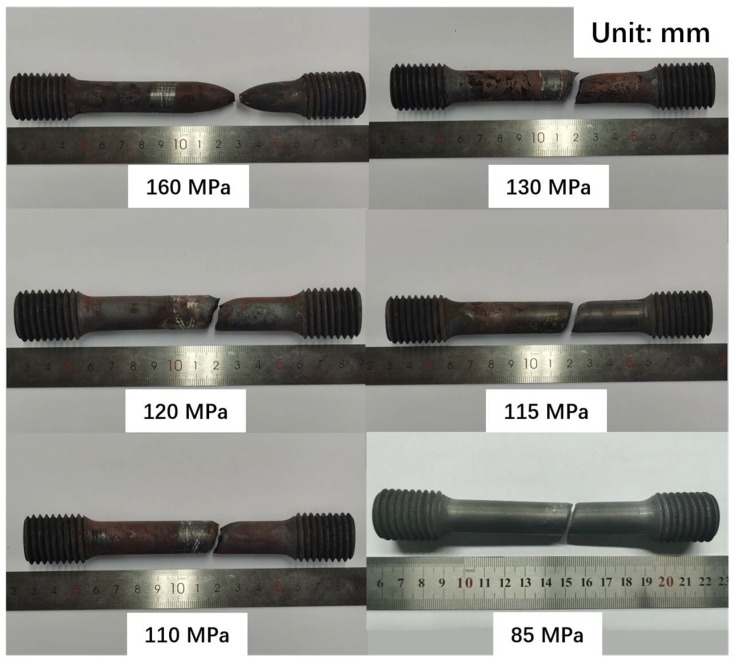
Some macro morphology of the samples after testing at 600 °C (unit: mm).

Creep tests are conducted under conditions of 550 °C, 575 °C, and 600 °C, with at least six stresses tested in each temperature group. After creep tests, the creep rupture data and fracture specimens of P91 steel welded joints were obtained. The results show that the fracture behavior of the joints can be divided into two types macroscopically (as shown in Figure 3, taking the test sample at 600 °C as a representative): 1. When the stress is relatively high, the specimen tends to break at the position away from the joint, with obvious plastic deformation and significant necking. 2. When the stress is relatively low, the fracture position is transferred from the BM to the HAZ, the fracture surface has no obvious deformation, and the fracture surface is about 75° to the loading direction, which is parallel to the fusion line (FL) and the HAZ.

**Figure 4:**
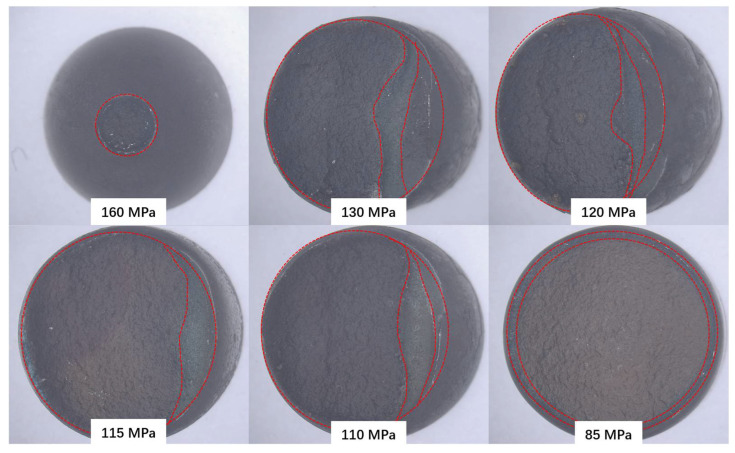
The macro morphology of the fracture surface under different stresses at 600 °C.

Figure 4 shows the macroscopic morphology of the fracture surface of the specimens under different stress conditions at 600 °C.

As can be seen from the figure, the failure behavior can be divided into three types from the fracture position and macroscopic morphology of the fracture surface: 1. The fracture located at the BM has obvious necking characteristics. 2. A mixed-fracture surface located in the HAZ with half macroscopic ductile deformation characteristics and half macroscopic brittle deformation characteristics. 3. The macroscopic brittle fracture surface that fractures in the HAZ.

The generation of three types of fractures is related to temperature and stress: when the temperature is constant, the type of fracture changes with a decrease in stress; when the applied stress is high enough, the BM with lower yield strength yields, and necking and fracture will occur in the subsequent time. As the stress decreases, the failure position of the joint shifts from the BM to the HAZ. At this time, half of the area shows macroscopic brittle fracture characteristics, while the other half experiences overload fracture, with obvious unilateral necking characteristics. As the stress further decreases, the area of macroscopic brittle cracking in the HAZ significantly increases, with only a small range of shear lip features remaining at the edge, thus forming a fracture surface feature of macroscopic complete brittle cracking. As the temperature increases, the stress corresponding to the transformation of this fracture type gradually decreases.

According to the macroscopic analysis of the creep fracture, the fracture behavior is divided into three types. In each fracture form, the characteristics of the same type of fracture are basically the same. In this paper, 600 °C-160 MPa, 600 °C-120 MPa, and 600 °C-85 MPa are taken as examples.

**Figure 5:**
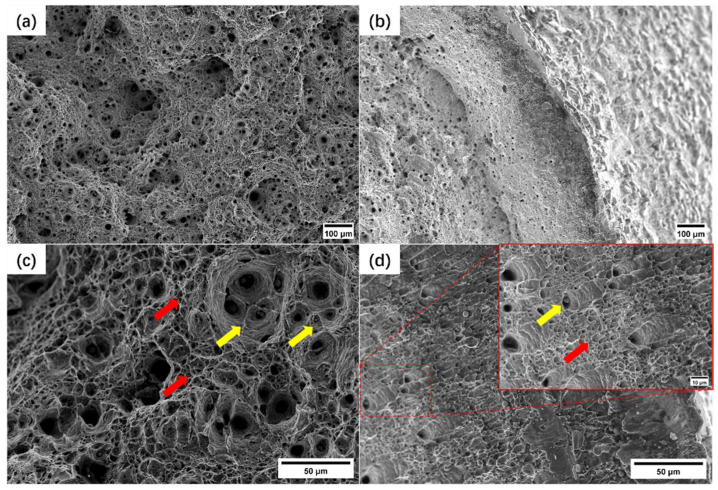
The fracture morphology of cracking at the BM: (**a**) fiber zone 100×; (**b**) shear lip 100×; (**c**) fiber zone 500×; (**d**) shear lip 500×. (The dimples with large diameter and depth are marked by yellow arrows and the small and shallow dimples are marked by red arrows).

Figure 5 shows the microstructure of the fracture surface under the test conditions of 600 °C-160 MPa.

Figure 5a,c show the characteristics of transgranular cracking under high stress. When the magnification reaches 500×, the middle part of the fracture surface presents the characteristics of many dimples with large depths (marked by yellow arrows), with many small and shallow dimples (marked by red arrows) gathered around them; the diameter of the large dimples is more than 10 μm, and spherical precipitate particles can be observed at the bottom of some dimples (marked by yellow arrows in Figure 5c). The adjacent large-sized dimples are internally necked during the long-term creep process and gradually merge to form cracks. The edge of the fracture is like a tensile fracture, with the characteristics of shear lips; the enlarged features are shown in Figure 5d, which are mainly shear-shaped dimples. The shear dimples are narrow and long, with a shallow depth, and a small number of large-sized dimples can be observed in the shear lip area marked by yellow arrows and red arrows, respectively. During the macroscopic necking process of the specimen, the large-sized dimples deflect under the action of triaxial stress, and the opening direction tilts outward.

**Figure 6:**
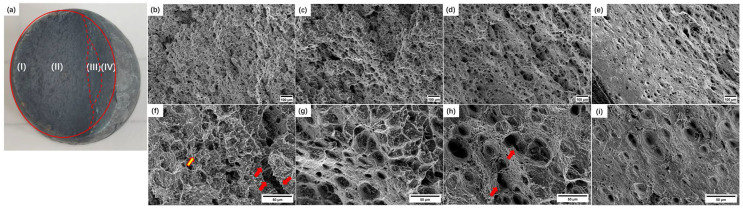
The fracture morphology of the HAZ with local necking: (**a**) macroscopic; (**b**) Region I 100×; (**c**) Region II 100×; (**d**) Region III 100×; (**e**) Region IV 100×; (**f**) Region I 500×; (**g**) Region II 500×; (**h**) Region III 500×; (**i**) Region IV 500×. (Oxidized dimples are marked by yellow symbols; the microcrack and merged voids are marked by red arrows).

Figure 6 shows the fracture surface characteristics of a creeped specimen with local necking.

As the applied stress decreases, the failure position changes from the BM to the HAZ. When the stress is relatively large, the fracture surface is divided into four areas (as shown in the red line in Figure 6a, named Regions I to IV), and the crack starts at Region I in the sample. At the current stage, the stress is relatively small; the microstructure is shown in Figure 6b,f, and the dimples are shallow. After cracking, slight oxidation occurs on the surface of the dimples, and the contour features are blurred, marked by yellow symbols in the figure; microcracks form in this area through the merging of adjacent voids with large sizes, as indicated by the red arrows in the figure. There are many precipitate particles in the ductile dimples, which are characterized by intergranular ductile fractures.

As the crack propagates, the net cross-sectional area of the specimen is reduced, and the actual stress of the specimen gradually increases, resulting in it being higher than the nominal stress. This is manifested as a significant increase in the depth of ductile dimples in the fracture surface, as shown in Figure 6c,g. In Region II, the characteristics of intergranular ductile cracking were maintained. Compared to the ductile dimples at Region I, the dimples at Region II are more uniform in size and depth, indicating that the stress and deformation are greater than those at Region I. As the cracks propagate further, the area occupied by Region I and Region II further increases, the remaining cross-sectional area of the specimen further decreases, and the stress on it significantly increases, exceeding the yield strength of the material; significant plastic deformation occurs in this portion, forming the same transgranular dimple characteristics as in the fiber zone under high-temperature tensile stress; the microstructure of Region III is same as that in Figure 5, with a large number of dimples, as shown in Figure 6d,h. When the actual stress is too high, the remaining uncracked part forms shear cracking, forming shear lip characteristics, as shown in Figure 6e,i.

**Figure 7:**
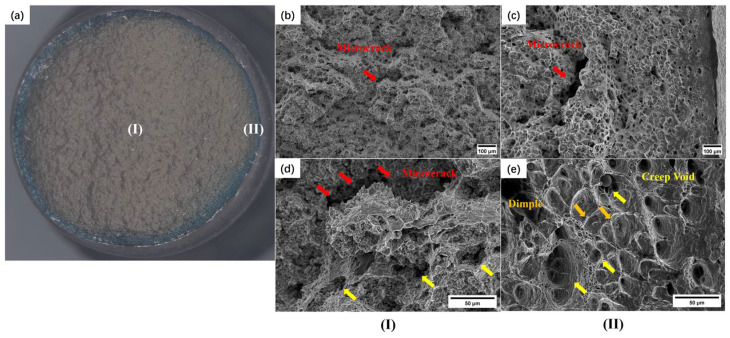
The fracture morphology of the HAZ without necking: (**a**) macroscopic; (**b**) center area 100×; (**c**) annular area 100×; (**d**) center area 500×; (**e**) annular area 500×.

When the stress further decreases, the third type of fracture will be formed, which macroscopically shows almost no elongation characteristics: only the fracture edge has very small necking, and the fracture surface is flat. Based on the oxidation degree analysis, the fracture surface can be divided into two areas: the inner gray circular area and the edge blue annular area. SEM observations were performed on each of the two regions, and the results are shown in Figure 7.

In the case of a completely brittle fracture, the fracture site is the gray area at the center, while the blue annular area is the last area where the overload fracture occurred. As can be seen from Figure 7c, the fracture surface has certain characteristics of brittle cracking along the grain at a large magnification, and there are a certain number of creep voids and microcracks at the grain boundary, which are typical creep fracture structures. As the crack propagates to the surrounding area, the net cross-sectional area of the specimen decreases significantly, the edge stress increases rapidly, and, finally, the microstructure of creep voids and dimples coexists due to overload.

### 3.3. Microstructure Characterization after Creep Test

**Figure 8:**
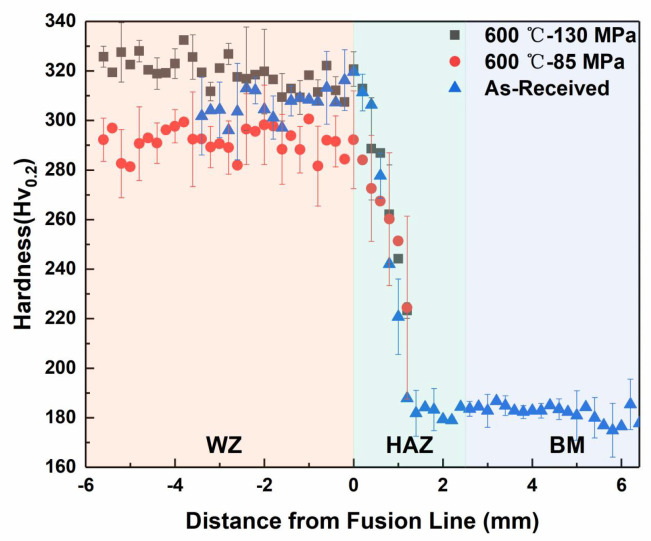
The hardness distribution of the cross-section at the fracture surface.

The hardness test was carried out on the fractured joint section, and its hardness distribution is shown in Figure 8.

Since there is no obvious plastic deformation when the joint is fractured, the hardness result can truly reflect the relationship between the hardness change and the fracture position. The blue triangle symbol in the diagram represents the hardness distribution of the joint before the creep test: the hardness of the WZ is about 320 Hv, the hardness of the HAZ gradients decreases towards the BM from the fusion line and decreases to a minimum in the over-tempering zone, and the BM hardness is about 185 Hv. The black symbols and the red symbols reflect the hardness distribution at the 600 °C-130 MPa and 600 °C-85 MPa fractures, respectively, and it can be seen from the data termination location that the fractures occur in the HAZ, and it is worth noting that the fracture locations are not concentrated in the over-tempering zone with the lowest hardness. The change in hardness in the WZ shows that the longer the service time, the more obvious the tendency of hardness to decrease during service, which is consistent with the hardness test results of the BM.

**Figure 9:**
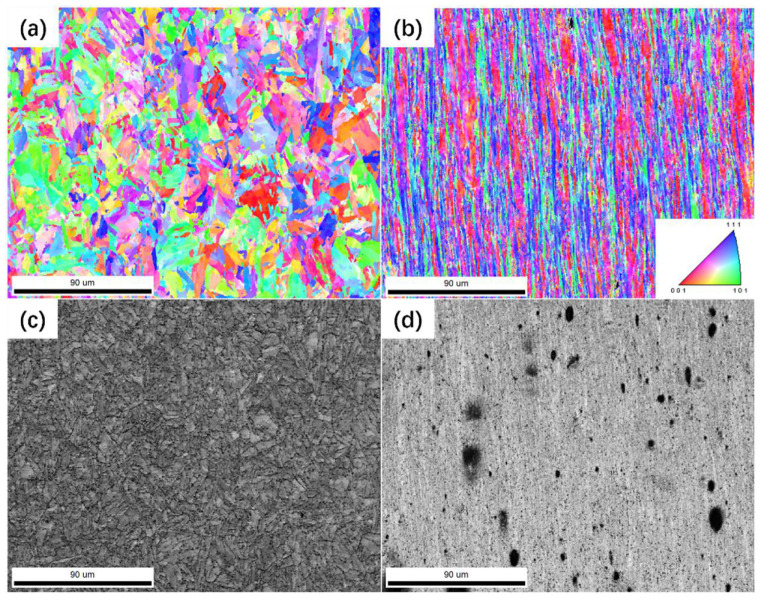
Overload fracture cross-section microstructure: (**a**) grain orientation map in the undeformed zone; (**b**) grain orientation map at the fracture surface; (**c**) undeformed microstructure SE image; (**d**) SE images of the microstructure near the fracture surface.

**Figure 10:**
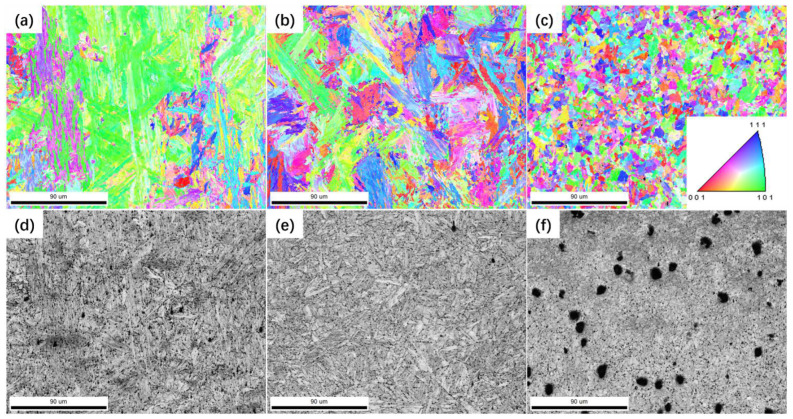
Morphology of creep fracture cross-section: (**a**) grain orientation in the WZ; (**b**) grain orientation in the CGHAZ; (**c**) grain orientation in the FGHAZ; (**d**) SE image of the WZ; (**e**) SE image of the CGHAZ; (**f**) SE image of the FGHAZ.

From the perspective of fracture position and fracture morphology, the failure of P91 steel joints can be divided into two categories: overload failure of the BM and creep failure of the FGHAZ; there are different damage mechanisms. Figure 9 and Figure 10 are the EBSD orientation and SE images of overload fracture cross-sections and creep failure fracture cross-sections, respectively.

Since the strength and hardness of the BM are much lower than those of welded joints, when the applied stress is large, it will cause the BM to yield first. Figure 9a,c show the undeformed BM structure, while Figure 9b,d are the structures near the fracture surface. Before the creep process, the original grains do not have obvious deformation or turning, basically maintaining the characteristics of martensite laths, and the orientation distribution is relatively uniform. During the creep process, many voids are generated and accumulated in the BM. When the external stress is high, the generation and coarsening of creep voids result in a decrease in the local net cross-sectional area, which in turn leads to an increase in the actual stress; when the stress is large enough, local yielding and necking will occur. The grains in the necked area undergo elongation and rotation under the larger stress to adapt to deformation, forming the microstructure characteristics shown in Figure 9b, which promotes the rapid occurrence of necking and causes the failure of the material.

When the stress decreases to the threshold stress, material yield is no longer the main cause of P91 steel welded joint failure. It can be seen from Figure 10 that the grain morphology of each region of the joint maintains the microstructure characteristics in the undeformed state, and the grains are randomly oriented without obvious deformation and rotation characteristics. Figure 10a,b,d,e show the microstructure characteristics of the WZ and the CGHAZ, both of which maintain the characteristics of coarse martensitic lathes, and some small voids can be observed in the SE images, showing irregular shapes. Figure 10c,f show the structure of the FGHAZ; the grains in this area are fine, and no obvious martensitic lathes are observed. It is worth noting that there are a large number of voids with a diameter of about 8 μm, and the spacing between a large number of voids is less than the average diameter. Figure 10c,f are located within 100 μm of the fracture edge.

### 3.4. Weld Joint Life Prediction

In current engineering practice, there are many methods used to describe the relationship between the strength, temperature, and life of heat-resistant steel materials or welded joints at operating temperatures, but the fracture form and location of the test materials are generally not considered. This is because the creep life prediction method of materials is generally based on reasonable extrapolation of short-term creep test data, and considering cost reasons, long-term experimental data are not or rarely used, so it is mostly manifested as a single-fracture mode. Common time–temperature parameter (TTP) methods include the Orr–Sherby–Dorn method [14], Larson–Miller method [15], Manson–Haferd method [16], etc., all of which are typical time parameter extrapolation methods based on experimental data. Taking the Larson–Miller method as an example, it can be described as follows:

(1) $$P=a_{0}+a_{1}log\sigma +a_{2}{log}^{2}\sigma +\cdots +a_{n}{log}^{n}\sigma +e^{n}$$

where *σ* is external stress, $a_{0}$, $a_{1}$, $a_{2}$ and $a_{n}$ are regression coefficients, and n is the order of the regression equation, generally taken as *n* = 2~4; $e^{n}$ is the error term, and the parameter *P* can be described as follows:

(2) $$P=T(C_{LM}+logt_{r})$$

where *T* is the test temperature in K; $t_{r}$ is the service time in hours; and $C_{LM}$ is the Larson–Miller constant. For P91 steel, the common values of $C_{LM}$ within the range of 550–750 °C are 20, 25, 33, 42, etc. For welded joints, according to the European Committee on Creep Cooperation (ECCC)’s high-temperature creep standard for P91 steel, the constant $C_{LM}$ value is 35. The constant $C_{LM}$ used in different studies varies greatly.

Based on the experimental results of P91 steel welded joints in this study, the constants in the Larson–Miller equation were fitted, and the relevant life prediction curves were plotted. The L-M constant $C_{LM}$ value obtained based on experimental results is 28.41, and the data fitting results are still within the generally recognized reasonable range of 20–36 [16]. To facilitate the comparison of the relationship between strength, temperature, and life, the $C_{LM}$ used in ECCC standard data [17] and the $C_{LM}$ used in joint fitting were normalized.

**Figure 11:**
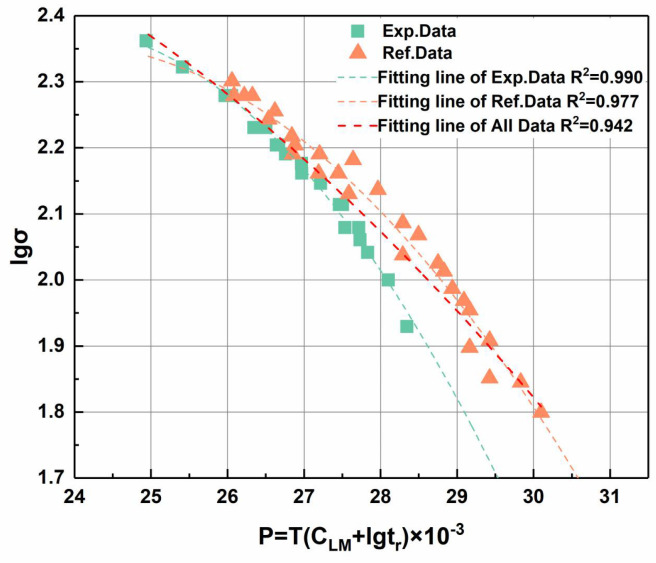
Relationships between the creep rupture life and applied stress of the P91 steel welded joints.

The results are shown in Figure 11, where the green rectangle symbol represents the measured data, and the orange triangle symbol represents the reference data. The reference data are the time–temperature creep strength data of P91 steel that was not put into use before the experiment. Creep data of P91 steel joints have not been found in other studies. The results indicate that the goodness-of-fit values of the fitting line are 0.990, 0.977, and 0.942, respectively.

Compared with the creep standard data of P91 steel, during the high-stress stage, the failure of the joint is manifested at the BM, and the creep strength is close to the data value in the ECCC standard [17]. As the stress gradually decreases, the effective service time of the test sample gradually decreases. When the stress at a specific temperature reaches a certain threshold, the failure mode and location change, transitioning from the BM to the HAZ, which is reflected in the Larson–Miller fitting curve in the form of an abrupt change in the slope of the curve. In addition, the change in the failure position significantly reduces the overall life of the joint, and there is no obvious plastic deformation in the HAZ before failure, making it difficult to prevent in advance.

**Figure 12:**
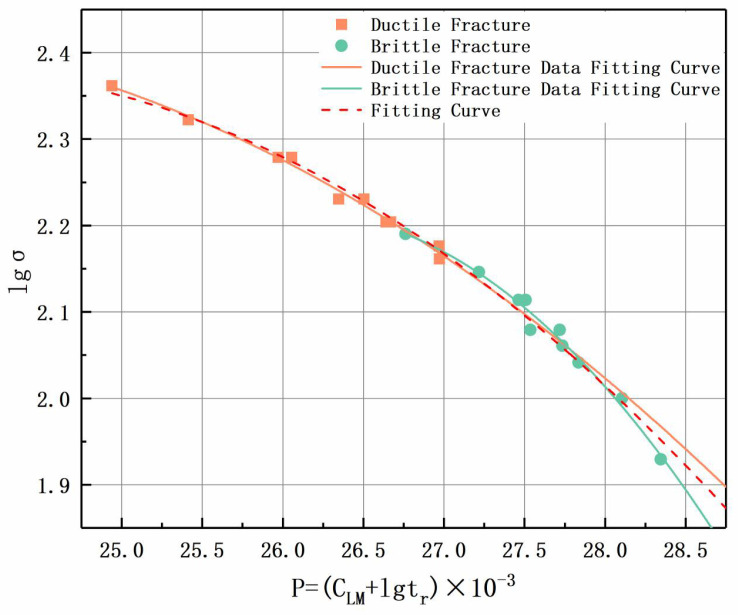
L-M curve fitting based on data of different fracture types.

To further illustrate the cause of the abrupt change in the slope of the curve in the test results, the fracture mode corresponding to the experimental data was considered when fitting the curve. As shown in Figure 12, after dividing the parameters into two sets, the fitting curve is divided into two curves.

The two curves describe the relationships between creep strength and life under different temperature and stress conditions. At the same time, there is an intersection between the two fitting curves, which corresponds to the location of the abrupt change in the slope of the curve in Figure 11. Through the reasonable extension of the fitting curves of the three sets of data, it can be seen that both the fitting curves obtained without considering the fracture mode (indicated by the red dotted line) and the curves fitted using only short-term creep test results (i.e., high-temperature and high-stress states) have a greater overfitting tendency than the actual results, which tend to increase further with an increase in temperature or a decrease in stress.

**Figure 13:**
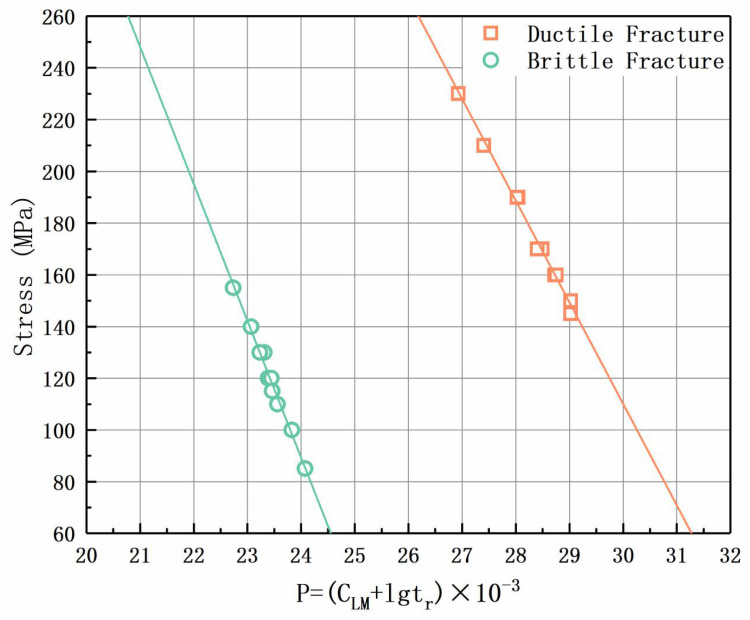
Fitting curve of BM and joint based on single test.

From Figure 11 and Figure 12, the strength of the high-stress set during the durability assessment of the joint is basically consistent with the test results of the BM. Based on the analysis of the fracture behavior of the high-stress section in the previous text, it can be concluded that during the testing process, the high-stress stage of the joint can reflect the creep strength of the BM. However, when the stress decreases, there is a turning point in the creep failure mode of the joint. Based on this, the $C_{LM}$ of both the base material and the joint can be obtained by only conducting joint creep testing and calculating the test results in curves. During this testing process, by fitting and analyzing the data, the calculation formulas for the creep strength of the BM and joint parts of the in-service joint components in subsequent service were obtained. Through simplification and linear calculation, the strength results were directly obtained, and the simplified results still have high accuracy. The simplified linear fitting result is shown in Figure 13, which has a high degree of fit.

(3) $$\sigma =a+b\times P=\left\{\begin{array}{ll} a_{1}+b_{1}T\left(30+\log t_{r}\right), & Broken\;from\;BM\\ a_{2}+b_{2}T\left(23+\log t_{r}\right), & Broken\;from\;FGHAZ \end{array}\right.$$

**Figure 14:**
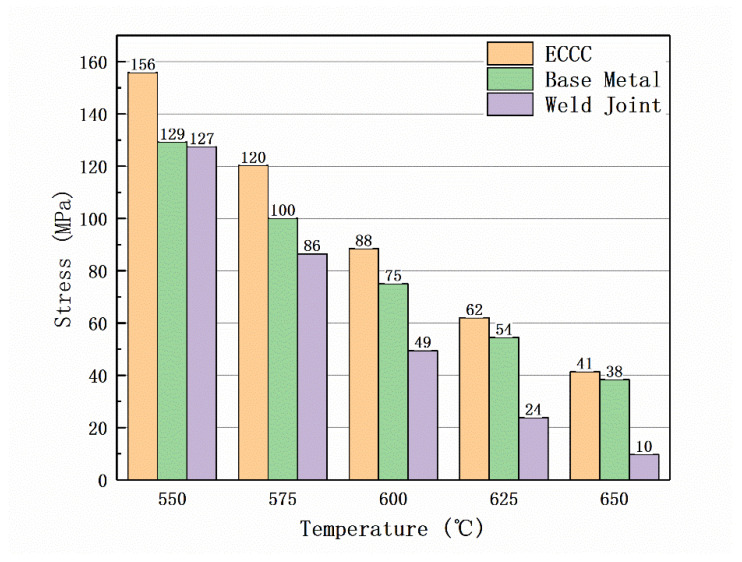
Comparison and prediction results of 10^5^ h strength and ECCC standard strength corresponding to different $C_{LM}$.

The results obtained from the double constant were extrapolated, and the predicted strength was compared with the 10^5^ h creep strength in the ECCC standard, as shown in Figure 14.

The ECCC result is the test result of the P91 steel without a service history, while the test result considers the cumulative 10^5^ h creep strength of its service history, resulting in a loss of substrate strength. As the working temperature increases, the strength of the joint and BM gradually decreases, and the difference in strength between the two also gradually expands. This also shows that it is necessary to classify the test results of the joints to accurately predict the strength of the complete part.

## 4. Conclusions

The creep fracture behavior of P91 heat-resistant steel and its welded joints, which had been in service for over 33,000 h, was studied at different temperatures. Some main conclusions are summarized as follows:

1. In the case of low hardness in the in-service P91 welded joint, its 10^5^ h creep strength at temperatures of 550 °C, 575 °C, and 600 °C is 119.7 MPa, 83.3 MPa, and 44.8 MPa, respectively. As the temperature increases, the strength of the in-service material is lower than the new joint material (550 °C, 575 °C, and 600 °C correspond to strengths of 119.8 MPa, 88 MPa, and 62.3 MPa, respectively [18]);
2. P91 steel welded joints are sensitive to temperature and load during service, and there are three main fracture modes: At 550 °C, ductile cracking of the BM occurs when the applied stress is greater than 160 MPa, and type IV cracking of the joint occurs when the applied stress is less than 160 MPa. At 575 °C, when the applied stress is greater than 140 MPa, it indicates ductile cracking of the BM. When the stress is less than 130 MPa, it indicates type IV cracking of the joint. At 600 °C, when the applied stress is greater than 130 MPa, it indicates ductile cracking of the BM. When the stress is less than 100 MPa, it indicates type IV cracking of the joint;
3. The L-M method with dual-constant $C_{LM}$ can simultaneously obtain the creep life and creep strength of P91 pipeline BM and joints, effectively reducing testing workload, avoiding early joint failure, and improving the safety of component service.

## Figures and Tables

**Table 1:** Chemical compositions of P91 steel welded joints (wt%).

Elements	C	Si	Mn	S	P	Cr	Mo	V	Nb
BM	0.093	0.29	0.50	0.012	0.0033	8.42	0.94	0.20	0.063
WZ	0.096	0.21	0.60	0.0075	0.0042	8.42	0.94	0.20	0.063
ASME-SA335P91	0.08~0.12	0.20~0.50	0.30~0.60	≤0.01	≤0.02	8.00~9.50	0.85~1.05	0.18~0.10	0.06~0.10

## Data Availability

The data presented in this study are available on request from the corresponding author due to project requirements, relevant data will not be publicly available until the end of the project.
